# Type 2 Diabetes Intensifies Nocturnal and Early Morning Circadian Autonomic Dysregulation After Ischaemic Stroke

**DOI:** 10.1155/jdr/4357436

**Published:** 2026-04-20

**Authors:** Jiann-Der Lee, Yen-Chu Huang, Tsong-Hai Lee, Meng Lee, Chuan-Pin Lee, Ya-Wen Kuo

**Affiliations:** ^1^ Department of Neurology, Chiayi Chang Gung Memorial Hospital, Chiayi, Taiwan, cgmh.org.tw; ^2^ College of Medicine, Chang Gung University, Taoyuan, Taiwan, cgu.edu.tw; ^3^ Department of Neurology, Linkou Chang Gung Memorial Hospital, Taoyuan, Taiwan, cgmh.org.tw; ^4^ Health Information and Epidemiology Laboratory, Chiayi Chang Gung Memorial Hospital, Chiayi, Taiwan, cgmh.org.tw; ^5^ Department of Nursing, Chang Gung University of Science and Technology, Chiayi Campus, Chiayi, Taiwan, cgust.edu.tw

**Keywords:** autonomic nervous system, circadian rhythm, diabetes, heart rate, ischaemic stroke

## Abstract

**Aims:**

Cardiac autonomic neuropathy (CAN) is a frequent but under‐recognised complication of diabetes that may exacerbate cardiovascular risk after stroke. This study aimed to characterise circadian patterns of heart rate (HR) and HR variability (HRV) in patients with acute ischaemic stroke, comparing those with and without type 2 diabetes.

**Methods:**

We conducted a retrospective case‐control study of 157 patients with acute ischaemic stroke who underwent 7‐day continuous electrocardiographic (ECG) monitoring (80 with type 2 diabetes, 77 without diabetes). Patients with paroxysmal atrial fibrillation, insufficient ECG data (<7 days), or obstructive sleep apnoea were excluded. HR and HRV indices (standard deviation of NN intervals [SDNNs], root mean square of successive differences [RMSSDs], pNN50, high‐frequency [HF], low‐frequency [LF] and LF/HF ratio) were derived from 5‐min segments. Circadian variation was analysed using generalised additive mixed models (GAMMs) adjusted for demographic, clinical and treatment factors.

**Results:**

Compared with patients without diabetes, those with type 2 diabetes exhibited persistently higher HR and lower HRV across both time‐ and frequency‐domain measures. Differences were most pronounced at night and in the early morning, indicating blunted autonomic fluctuations despite a preserved HR rhythm. Among patients with diabetes, poorer glycaemic control (glycated haemoglobin [HbA1c] > 7% [53 mmol/mol]) was associated with higher HR and lower HRV, particularly during nocturnal hours, supporting a dose–response relationship.

**Conclusions:**

Type 2 diabetes is associated with attenuated circadian autonomic regulation after ischaemic stroke, characterised by elevated HR and reduced HRV, most evident during sleep and early morning periods. Continuous ECG monitoring may facilitate early detection of nocturnal autonomic dysfunction, and interventions that enhance parasympathetic tone—such as improved glycaemic control and potentially cholinergic modulation—warrant further investigation to mitigate post‐stroke cardiovascular risk.

## 1. Introduction

Diabetes is a well‐established independent risk factor for stroke, with studies showing higher incidence and mortality rates among affected individuals [1, 2]. It causes a range of complications involving multiple organ systems, among which cardiac autonomic neuropathy (CAN) is well‐recognised yet often underdiagnosed. CAN disrupts autonomic regulation of cardiac function and is associated with an increased risk of cardiovascular events and mortality [3]. However, the circadian profile of autonomic function in diabetic patients during the acute post‐stroke phase remains poorly characterised compared with that in non‐diabetic stroke patients.

Heart rate (HR) and HR variability (HRV) are non‐invasive, valuable tools for assessing cardiac autonomic function and identifying individuals at risk for CAN [4, 5]. Diabetes exerts profound and detrimental effects on the circadian regulation of HR and HRV, primarily through the development of CAN [6, 7]. Elevated resting HR, blunted nocturnal HR dips and a generalised reduction in HRV parameters are consistently observed in individuals with diabetes, reflecting widespread impairment of the autonomic nervous system [8]. Notably, diabetes has been reported with increased frequency among patients presenting with wake‐up stroke [9, 10], which implies that diabetes‐related disturbances in circadian regulation may primarily manifest during sleep or in the early morning hours before awakening.

Chronic hyperglycaemia and longer disease duration have been identified as key drivers of this autonomic dysfunction [11]. Early detection of these abnormalities through accessible tools like 24‐h electrocardiographic (ECG) monitoring and HRV analysis is crucial for risk stratification. However, few studies have employed long‐term ECG monitoring to evaluate the impact of type 2 diabetes on circadian HR and HRV patterns in patients with ischaemic stroke.

In this study, we aimed to investigate the circadian rhythms of HR and HRV in patients with ischaemic stroke using continuous 7‐day ECG monitoring and to assess whether type 2 diabetes and glycaemic control status are associated with alterations in these parameters. By characterising time‐dependent changes in autonomic activity, we sought to clarify the clinical significance of diabetes‐related autonomic dysfunction in the post‐stroke setting. We hypothesised that type 2 diabetes attenuates circadian autonomic fluctuations, particularly during nocturnal and early morning periods when parasympathetic dominance is expected. Understanding these patterns may help identify high‐risk periods for cardiovascular events and guide autonomic‐targeted interventions after stroke.

## 2. Materials and Methods

### 2.1. Study Design and Population

This retrospective case‐control study represents a secondary analysis based on data collected from a prior investigation titled Using 14‐Day Continuous Electrocardiography Patch Monitoring to Detect Paroxysmal Atrial Fibrillation After Stroke (NCT05218473). The original research, ongoing since February 2022, is a prospective observational study designed to assess the effectiveness of continuous ECG patch monitoring in identifying paroxysmal atrial fibrillation among patients with acute ischaemic stroke [12].

For this secondary analysis, additional exclusion criteria were applied to the original cohort. Patients were excluded if they met any of the following conditions: (1) paroxysmal atrial fibrillation was detected using the 14‐day ECG patch monitor; (2) fewer than 7 days of usable ECG data were recorded; (3) the patch monitor was applied more than 30 days after stroke; or (4) a known history of obstructive sleep apnoea was present. The selection process is illustrated in Supporting Information 1: Figure S1.

### 2.2. HRV Analysis

HRV assessment was conducted using a 14‐day continuous ECG patch monitor (EZYPRO, UG01, Sigknow Biomedical Co., Ltd, Taipei, Taiwan) operating at 256 Hz sampling frequency. To ensure uniform data quality and minimise variability arising from patch detachment, skin irritation and signal noise associated with prolonged wear time, only the first 7 days of monitoring were included in the analysis. This approach also provided a consistent observation window across participants. Continuous ECG data underwent processing to extract RR intervals, which were subsequently validated through manual inspection by trained technicians. An automated filtering algorithm was applied to identify artefacts and ectopic beats, followed by manual correction to ensure data quality.

Time‐domain HRV parameters were computed from 5‐min ECG segments, encompassing standard deviation of NN intervals (SDNNs), root mean square of successive differences (RMSSD) between RR intervals and pNN50 (percentage of consecutive normal RR intervals differing by >50 milliseconds [ms]). Frequency‐domain metrics were derived using the Fast Fourier Transform, quantifying low‐frequency (LF; 0.04–0.15 Hz) and high‐frequency (HF; 0.15–0.4 Hz) power, as well as their ratio (LF/HF). All analytical procedures conformed to standards established by the Task Force of the European Society of Cardiology and the North American Society of Pacing and Electrophysiology [13].

To visualise the data, we analysed trends in HR and HRV parameters by dividing participants into groups with and without type 2 diabetes. For each group, the mean and standard error of HR and HRV parameters were calculated at 5‐min intervals. We then generated smoothed curves with 95% confidence intervals (CIs) to illustrate the 24‐h patterns of HR and HRV fluctuations.

### 2.3. Statistics

Quantitative variables are presented as means (standard deviations), and categorical variables as counts (percentages). To assess differences between patients with and without diabetes, the Student’s *t*‐test and chi‐squared test were used for continuous and categorical variables, respectively.

To assess the variations in HR and HRV parameters between patients with and without diabetes over the 24‐h period, generalised additive mixed models (GAMMs) were utilised [14]. The model formula was specified as:

HR or HRV parameters∼μ+stime, by= diabetes+β1diabetes+β2other fixed−effect variables+ random effects.

Temporal dynamics were modelled using circular smoothing splines, with diabetes‐by‐time interaction terms included to assess group‐specific differences. Repeated measures were addressed by specifying random intercepts for each participant in combination with a first‐order autoregressive correlation structure. Covariates were selected a priori based on clinical relevance and established associations with autonomic function, rather than by data‐driven variable selection, to reduce model instability and selective overfitting. Multicollinearity among adjustment covariates was assessed using the variance inflation factor diagnostics, and no evidence of problematic collinearity was identified. Adjustments were made for potential confounding factors, including age, sex, hypertension, coronary artery disease, heart failure, wake‐up stroke, stroke severity and location, body mass index, relevant laboratory values (creatinine, alanine aminotransferase, total cholesterol, triglycerides, white blood cell count and haemoglobin) and medications (beta‐blockers and non‐dihydropyridine calcium channel blockers). The overall effect of diabetes was assessed by combining the time‐dependent smooth terms with the parametric diabetes effect, and 95% CIs were derived from the model estimates. In addition to the fully adjusted models, descriptive and crude (unadjusted) analyses were conducted to evaluate the robustness of the observed temporal patterns. Similar GAMMs, as described above, were applied to evaluate the impact of glycaemic control (glycated haemoglobin [HbA1c] > 7% [53 mmol/mol] vs. ≤ 7% [53 mmol/mol]) on 24‐h HR and HRV rhythms among ischaemic stroke patients with type 2 diabetes. All analyses were conducted using R (Version 4.4.1; R Foundation for Statistical Computing, Vienna, Austria), with GAMMs fitted via the mgcv package (https://cran.r-project.org/web/packages/mgcv/index.html).

The study was conducted in accordance with the Declaration of Helsinki. The study protocol was approved by the institutional review board, and all participants provided written informed consent.

## 3. Results

### 3.1. Baseline Characteristics

A total of 157 adult patients with ischaemic stroke were included in the analysis (77 without diabetes and 80 with type 2 diabetes). Baseline demographic characteristics, vascular risk factors, laboratory findings, stroke features and medication use are summarised in Table 1. Stroke‐related clinical parameters, including stroke severity category based on NIHSS grouping and stroke lesion location, were comparable between groups. The comparison revealed significant differences between groups in sex, history of hypertension and coronary artery disease, and levels of HbA1c, creatinine and total cholesterol (Table 1). Patients with type 2 diabetes had a higher mean HR (75.89 ± 14.20 vs. 71.89 ± 14.76 beats per minute [bpm], *p*  < 0.001) and lower HRV, as evidenced by lower SDNN (34.06 ± 26.07 vs. 44.67 ± 35.62 ms, *p*  < 0.001), RMSSD (19.78 ± 21.12 vs. 27.35 ± 47.88 ms, *p*  < 0.001) and pNN50 (2.17% ± 5.05% vs. 2.92% ± 6.11%, *p*  < 0.001). They also exhibited lower HF (38.50 ± 135.03 vs. 119.48 ± 736.67 ms^2^, *p*  < 0.001), LF (83.05 ± 235.43 vs. 136.88 ± 318.94 ms^2^, *p*  < 0.001) and LF/HF ratio (4.39 ± 4.75 vs. 4.43 ± 4.42, *p* = 0.026) (Table 2), indicating altered autonomic balance. These baseline differences indicate that type 2 diabetes is associated with impaired autonomic regulation before considering circadian variation and relevant covariates.

**Table 1:** Baseline characteristics of the study population.

Clinical background	Without diabetes (*n* = 77	With type 2 diabetes (*n* = 80)	*p* value
Male *n* (%)	46 (59.74)	62 (77.50)	0.02
Age (years), mean (SD)	64.44 (16.99)	65.14 (15.34)	0.96
Medical history *n* (%)			
Hypertension	50 (64.94)	74 (92.50)	<0.01
Coronary artery disease	2 (2.60)	10 (12.50)	0.02
Congestive heart failure	2 (2.60)	2 (2.50)	0.97
Stroke severity *n* (%)	—	—	0.12
NIHSS 0–4	49 (63.64)	49 (61.25)	—
NIHSS 5–15	18 (23.38)	27 (33.75)	—
NIHSS > 15	10 (12.99)	4 (5.00)	—
Stroke location, *n* (%)	—	—	0.91
Right hemisphere	26 (33.77)	27 (33.75)	—
Left hemisphere	30 (38.96)	29 (36.25)	—
Bilateral hemisphere	6 (7.79)	5 (6.25)	—
Posterior circulation	15 (19.48)	19 (23.75)	—
Wake‐up stroke, *n* (%)	4 (5.19)	5 (6.25)	0.78
Laboratory data, mean (SD)			
Glycated haemoglobin (%)	5.71 (0.36)	7.62 (1.89)	<0.01
Creatinine (μmol/L)	79.88 (33.24)	101.39 (62.79)	0.008
Alanine aminotransferase (U/L)	25.30 (16.38)	22.94 (14.35)	0.34
Total cholesterol (mmol/L)	4.66 (0.90)	4.17 (1.07)	<0.01
Triglycerides (mmol/L)	1.37 (0.89)	1.71 (1.48)	0.10
White blood cell count (10^9^/L)	8.73 (2.86)	8.30 (2.97)	0.36
Haemoglobin (g/L)	137.08 (25.87)	135.79 (18.72)	0.72
Medication, *n* (%)			
Beta‐blocker	8 (10.39)	7 (8.75)	0.73
Non‐dihydropyridine CCB	3 (3.90)	3 (3.75)	0.96
Interval between stroke onset and testing (days), mean (SD)	8.12 (6.68)	6.90 (5.24)	0.20

Abbreviations: CCB, calcium channel blocker; n, number; NIHSS, National Institute of Health Stroke Scale; Q, quartile; SD, standard deviation.

**Table 2:** Comparison of mean heart rate and heart rate variability parameters between ischaemic stroke patients with and without diabetes.

Heart rate variability parameters Mean (standard deviation)	Without diabetes (*n* = 77)	With type 2 diabetes (*n* = 80)	*p* value
Heart rate (bpm)	71.89 (14.76)	75.89 (14.20)	<0.001
SDNN (ms)	44.67 (35.62)	34.06 (26.07)	<0.001
RMSSD (ms)	27.35 (47.88)	19.78 (21.12)	<0.001
pNN50 (%)	2.92 (6.11)	2.17 (5.05)	<0.001
HF (ms^2^)	119.48 (736.67)	38.50 (135.03)	<0.001
LF (ms^2^)	136.88 (318.94)	83.05 (235.43)	<0.001
LF/HF	4.43 (4.42)	4.39 (4.75)	0.026

Abbreviations: bpm, beats per minute; ms, millisecond.

### 3.2. Circadian Variation in HR Between Ischaemic Stroke Patients With and Without Diabetes

As shown in Figure 1A, both groups maintained a similar 24‐h HR rhythm, with values lowest around 04:00, peaking between 08 : 00 and 18 : 00, and declining thereafter. However, patients with type 2 diabetes consistently exhibited higher HRs than those without diabetes across the entire day. The difference was most pronounced between 0 :00 and 05:00, when the 95% CIs did not overlap for most time points, indicating statistical significance. These results suggest that although patients with type 2 diabetes had persistently elevated HRs, their overall diurnal HR pattern remained preserved after stroke.

Figure 1Circadian variation in HR between ischaemic stroke patients with and without diabetes. (A) Smoothed visualisation of HR trends in ischaemic stroke patients with and without diabetes. Mean HRs (solid lines) were calculated at 5‐min intervals, with standard error indicated by shaded areas representing 95% CIs. This descriptive analysis highlights circadian changes in HR for each group over the 24‐h period. (B) Statistical evaluation of differences in HRs between ischaemic stroke patients with and without diabetes using generalised additive mixed models. Circular smoothing splines were used to model time‐varying effects, and the analysis was adjusted for covariates including age, sex, hypertension, coronary artery disease, heart failure, wake‐up stroke, stroke severity and location, body mass index, relevant laboratory values and medications. Solid lines depict predicted HR differences, with 95% CIs shown as shaded bands. CI, confidence interval; HR, heart rate.

**Figure:**
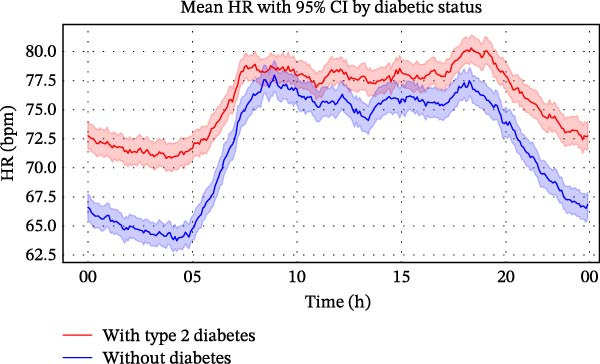
(A)

**Figure:**
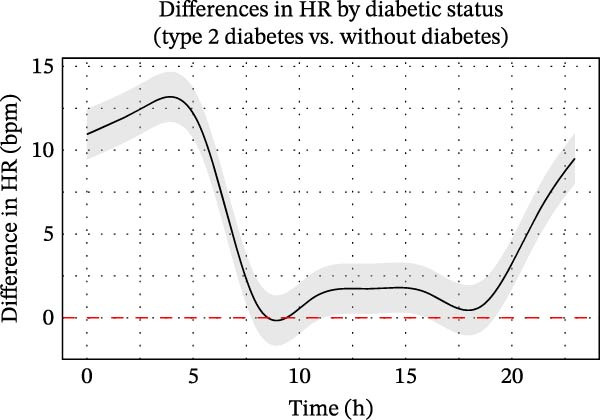
(B)

As illustrated in Figure 1B, the GAMM analysis, adjusted for time‐varying effects and relevant covariates, showed that the diabetes‐related elevation in HR peaked at ~13 bpm around 04:00, declined to near zero by 09:00, and rose again in the evening, reaching about 11 bpm by midnight. This time‐dependent pattern supports an independent association between diabetes and elevated HR, particularly during nighttime and early morning hours.

These findings support the hypothesis that diabetes modifies circadian autonomic activity, particularly by elevating HR during nocturnal and early morning periods when parasympathetic dominance is expected.

### 3.3. Circadian Variation in Time‐Domain HRV Parameters Between Ischaemic Stroke Patients With and Without Diabetes

**Figure 2:**
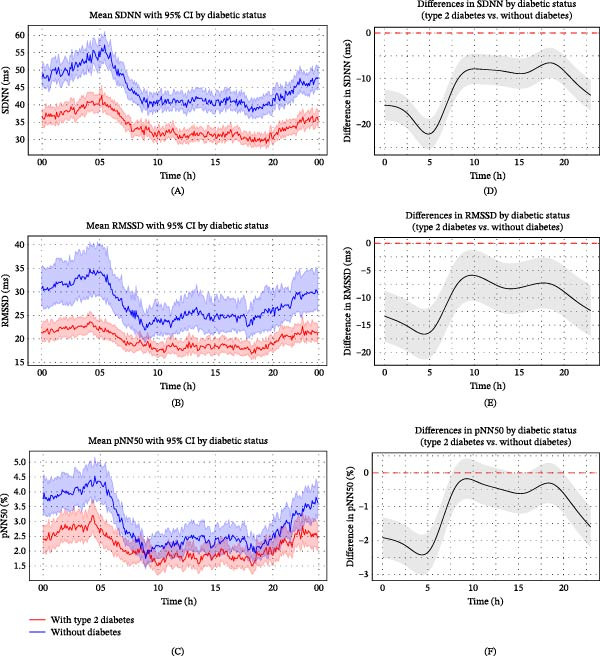
Circadian variation in time‐domain HRV parameters between ischaemic stroke patients with and without diabetes. (A–C) Trends in SDNN (A), RMSSD (B) and pNN50 (C) over time for ischaemic stroke patients with and without diabetes. Time domain parameters of HRV (solid lines) were calculated at 5‐min intervals, with standard error indicated by shaded areas representing 95% confidence intervals. (D–F) Statistical evaluation of differences in SDNN (D), RMSSD (E) and pNN50 (F) between ischaemic stroke patients with and without diabetes using generalised additive mixed models. Circular smoothing splines were used to model time‐varying effects, and the analysis was adjusted for covariates including age, sex, hypertension, coronary artery disease, heart failure, wake‐up stroke, stroke severity and location, body mass index, relevant laboratory values and medications. Solid lines depict predicted HRV differences, with 95% CIs shown as shaded bands. CI, confidence interval; HRV, heart rate variability; ms, millisecond.

Patients without diabetes consistently demonstrated higher time‐domain HRV parameters—SDNN, RMSSD and pNN50—compared to those with type 2 diabetes. In patients without diabetes, SDNN peaked at ~57 ms around 05:00–06:00, declined to 38–42 ms between 09:00 and 20:00, and rose again to about 45 ms in the late evening. In contrast, patients with type 2 diabetes exhibited lower and flatter SDNN values (30–43 ms), peaking at ~43 ms in the early morning and declining to around 37 ms by evening (Figure 2A).

RMSSD and pNN50 were also consistently higher in patients without diabetes, reflecting greater parasympathetic activity. Although the pNN50 gap narrowed during 08:00–16:00 (with partial CI overlap), RMSSD remained significantly different across nearly all time points. Both parameters peaked around 04:00–05:00 and were lowest from 08:00 to 18:00. Diurnal variation was more pronounced in patients without diabetes, while patients with type 2 diabetes exhibited blunted fluctuations (Figure 2B,C), suggesting altered autonomic regulation.

GAMM analyses demonstrated significantly lower SDNN, RMSSD and pNN50 in the diabetic group, with the most pronounced differences observed during the early morning hours (Figure 2D–F). These differences peaked around 05:00, diminished in the late morning and re‐emerged in the evening. After adjusting for confounders, diabetes remained independently associated with reduced and time‐dependent suppression of HRV, most notably during the night and early morning. This attenuation of HRV rhythms supports the notion that diabetes is associated with reduced circadian autonomic flexibility following stroke.

### 3.4. Circadian Variation in Frequency‐Domain Parameters of HRV Between Ischaemic Stroke Patients With and Without Diabetes

**Figure 3:**
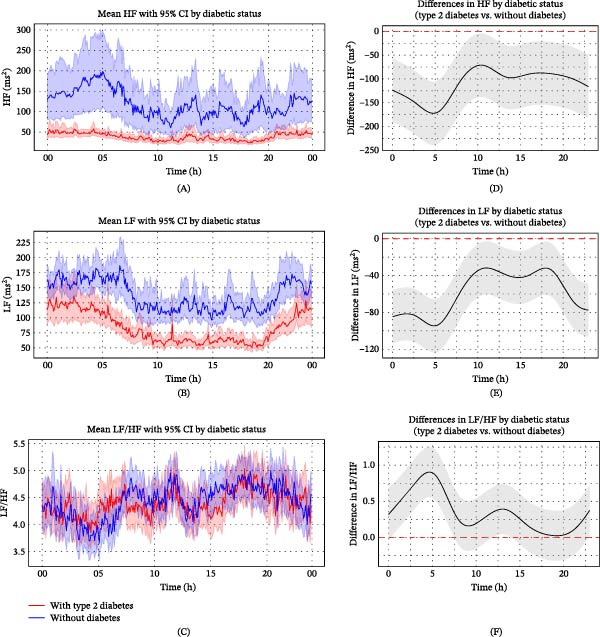
Circadian variation in frequency‐domain HRV parameters between ischaemic stroke patients with and without diabetes. (A–C) Trends in HF (A), LF (B) and LF/HF (C) over time for ischaemic stroke patients with and without diabetes. Frequency domain parameters of HRV (solid lines) were calculated at 5‐min intervals, with standard error indicated by shaded areas representing 95% CIs. (D–F) Statistical evaluation of differences in HF (D), LF (E) and LF/HF (F) between ischaemic stroke patients with and without diabetes using generalised additive mixed models. Circular smoothing splines were used to model time‐varying effects, and the analysis was adjusted for covariates including age, sex, hypertension, coronary artery disease, heart failure, wake‐up stroke, stroke severity and location, body mass index, relevant laboratory values and medications. Solid lines depict predicted HRV differences, with 95% CIs shown as shaded bands. CI, confidence interval; HRV, heart rate variability; ms, millisecond.

Frequency‐domain HRV analysis revealed distinct circadian patterns and significant differences between patients with and without diabetes. In patients without diabetes, HF power exhibited marked circadian variation, peaking at ~200 ms^2^ around 05:00 and declining to 60–142 ms^2^ during the day. In contrast, patients with type 2 diabetes exhibited persistently lower HF power (24–60 ms^2^) with minimal circadian fluctuation (Figure 3A). LF power showed a similar pattern: patients without diabetes demonstrated nocturnal elevations (~150–190 ms^2^) and daytime reductions (~100–140 ms^2^), whereas those with type 2 diabetes maintained consistently lower values (~50–140 ms^2^) with an attenuated circadian rhythm (Figure 3B). The LF/HF ratio showed no substantial group difference, with overlapping CIs and similar temporal trends (Figure 3C).

GAMM analysis, adjusted for time‐varying effects and relevant covariates, confirmed that patients with type 2 diabetes consistently had lower HF and LF power across the 24‐h period, with CIs for group differences remaining below zero. HF power differences were greatest around 05:00 and peaked near 10:00 (Figure 3D), while LF showed a similar temporal pattern (Figure 3E). The LF/HF ratio was generally higher in the diabetic group, peaking around 05:00, suggesting a shift toward sympathetic dominance or diminished parasympathetic modulation (Figure 3F). These results further support the hypothesis that type 2 diabetes attenuates circadian autonomic oscillations, particularly during periods of expected parasympathetic predominance.

To further evaluate the robustness of these findings, we compared the fully adjusted GAMM results with the descriptive smoothed curves derived directly from the raw, unadjusted HR and HRV data (Figures 1A and 2A–C). The overall circadian patterns and between‐group differences were highly consistent across descriptive and adjusted analyses, including the nocturnal and early‐morning divergence between groups. In addition, crude models without covariate adjustment showed directionally similar temporal patterns (Supporting Information 2: Figure S2). The concordance of results across unadjusted descriptive analyses, crude models and fully adjusted GAMMs supports the stability of the observed associations and reduces the likelihood that the findings are attributable to model specification or overadjustment.

Furthermore, stratified analyses by stroke recovery stage and stroke severity category demonstrated similar circadian trajectories across strata (Supporting Information 3: Figure S3 and Supporting Information 4: Figure S4), further supporting the robustness of the observed circadian attenuation pattern.

### 3.5. Circadian Variation in HR and HRV Parameters According to Glycaemic Control Status in Ischaemic Stroke Patients With Type 2 Diabetes

**Figure 5:**
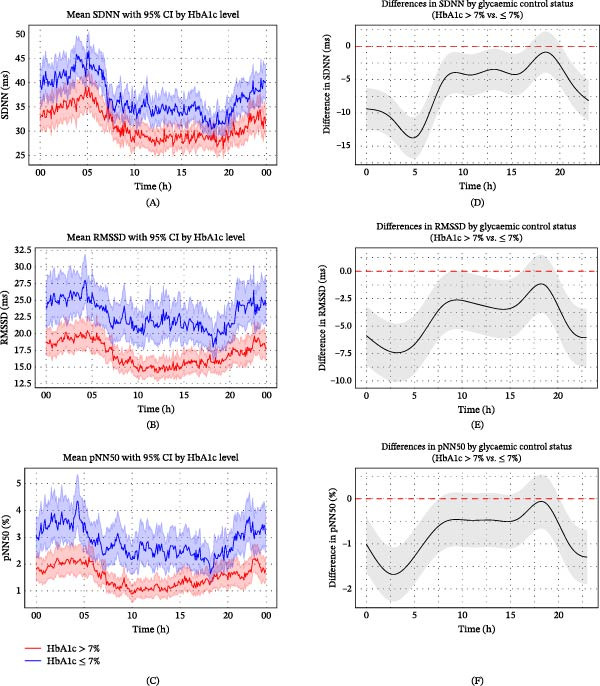
Circadian variation in time‐domain HRV parameters according to glycaemic control status in ischaemic stroke patients with type 2 diabetes. (A–C) Trends in SDNN (A), RMSSD (B) and pNN50 (C) over time according to glycaemic control status in ischaemic stroke patients with type 2 diabetes. Time domain parameters of HRV (solid lines) were calculated at 5‐min intervals, with standard error indicated by shaded areas representing 95% confidence intervals. (D–F) Statistical evaluation of differences in HF (D), LF (E) and LF/HF (F) according to glycaemic control status in ischaemic stroke patients with type 2 diabetes using generalised additive mixed models. Circular smoothing splines were used to model time‐varying effects, and the analysis was adjusted for covariates including age, sex, hypertension, coronary artery disease, heart failure, wake‐up stroke, stroke severity and location, body mass index, relevant laboratory values and medications. Solid lines depict predicted HRV differences, with 95% CIs shown as shaded bands. CI, confidence interval; HbA1c, glycated haemoglobin; HRV, heart rate variability; ms, millisecond.

**Figure 6:**
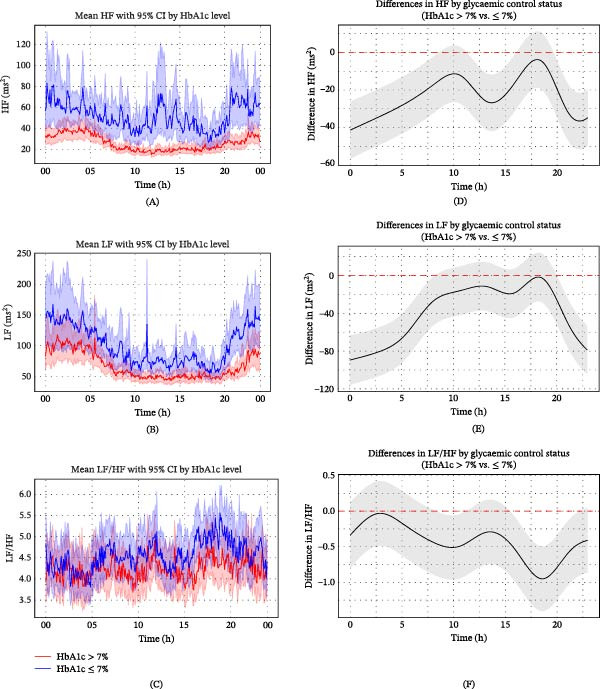
Circadian variation in frequency‐domain HRV parameters according to glycaemic control status in ischaemic stroke patients with type 2 diabetes. (A–C) Trends in HF (A), LF (B) and LF/HF (C) over time according to glycaemic control status in ischaemic stroke patients with type 2 diabetes. Frequency domain parameters of HRV (solid lines) were calculated at 5‐min intervals, with standard error indicated by shaded areas representing 95% confidence intervals. (D–F) Statistical evaluation of differences in HF (D), LF (E) and LF/HF (F) according to glycaemic control status in ischaemic stroke patients with type 2 diabetes using generalised additive mixed models. Circular smoothing splines were used to model time‐varying effects, and the analysis was adjusted for covariates including age, sex, hypertension, coronary artery disease, heart failure, wake‐up stroke, stroke severity and location, body mass index, relevant laboratory values and medications. Solid lines depict predicted HRV differences, with 95% CIs shown as shaded bands. CI, confidence interval; HbA1c, glycated haemoglobin; HRV, heart rate variability; ms, millisecond.

Among patients with type 2 diabetes, those with poor glycaemic control (HbA1c > 7%) exhibited higher HRs and lower HRV parameters compared to those with HbA1c ≤ 7%, most notably during the nighttime and early morning hours. The temporal profiles and directional trends of these differences closely resembled those observed between patients with and without diabetes, although the magnitude was smaller, suggesting a dose–response relationship between glycaemic burden and autonomic dysregulation (Figures 4–6). This relationship between glycaemic control and autonomic dysregulation reinforces the hypothesis that metabolic burden exacerbates circadian autonomic attenuation in type 2 diabetes after ischaemic stroke.

Figure 4Circadian variation in HR according to glycaemic control status in ischaemic stroke patients with type 2 diabetes. (A) Smoothed visualisation of HR trends according to glycaemic control status in ischaemic stroke patients with type 2 diabetes. Mean HRs (solid lines) were calculated at 5‐min intervals, with the standard error indicated by shaded areas representing 95% CIs. This descriptive analysis highlights circadian changes in HR for each group over the 24‐h period. (B) Statistical evaluation of differences in HRs according to glycaemic control status in ischaemic stroke patients with type 2 diabetes using generalised additive mixed models. Circular smoothing splines were used to model time‐varying effects, and the analysis was adjusted for covariates including age, sex, hypertension, coronary artery disease, heart failure, wake‐up stroke, stroke severity and location, body mass index, relevant laboratory values and medications. Solid lines depict predicted HR differences, with 95% CIs shown as shaded bands. CI, confidence interval; HbA1c, glycated haemoglobin; HR, heart rate.

**Figure:**
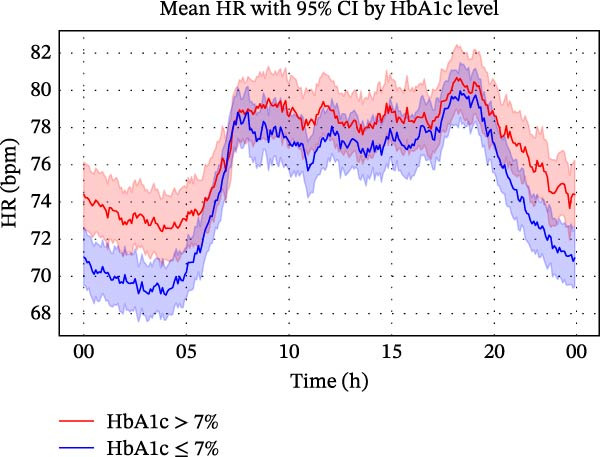
(A)

**Figure:**
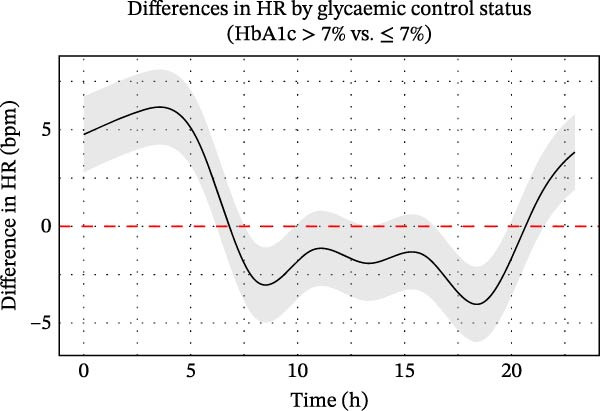
(B)

## 4. Discussion

This study demonstrates that type 2 diabetes disrupts circadian autonomic regulation after ischaemic stroke, particularly during the nocturnal and early morning periods when parasympathetic dominance is physiologically expected. Patients with type 2 diabetes exhibited persistently elevated HRs and markedly reduced HRV across both time‐ and frequency‐domain measures than those without diabetes. Despite maintaining an overall circadian rhythm of HR, they showed blunted autonomic fluctuations, most evident at night and before awakening. Moreover, poorer glycaemic control was associated with greater autonomic dysfunction, supporting a dose–response relationship. Together, these findings support our hypothesis that diabetes is associated with attenuated circadian autonomic flexibility after ischaemic stroke and suggest that nocturnal autonomic dysregulation may merit further investigation as a potential therapeutic target.

Diabetes increases stroke risk through mechanisms such as endothelial dysfunction, arterial stiffness, inflammation and cardiac abnormalities, with impaired nitric oxide signalling and heightened inflammatory activity contributing to atherosclerosis and cardiovascular complications [15]. Beyond these mechanisms, CAN has been independently associated with a higher risk of stroke in a large cohort of adults with type 2 diabetes [16]. CAN is a common yet underdiagnosed complication of diabetes, arising from nerve and vascular damage and leading to impaired HR control and abnormal vascular responses [17]. A reduction in HRV is among the earliest subclinical signs of CAN, typically beginning with parasympathetic impairment before progressing to sympathetic dysfunction [18]. CAN also profoundly disrupts the circadian rhythm of HR and HRV in diabetic patients, as evidenced by a reversal of normal day–night modulation and impaired sympathovagal balance. These alterations in autonomic regulation may contribute to the increased risk of nocturnal arrhythmias and adverse cardiac outcomes—including wake‐up stroke—observed in diabetic patients [9, 10, 12, 19].

Our study showed that ischaemic stroke patients with type 2 diabetes exhibited consistently higher HR and lower HRV indices—particularly SDNN, RMSSD, pNN50 and HF—than those without diabetes, with differences most pronounced during the night and early morning hours. These differences were less evident during the day. Parasympathetic activity typically peaks at night, whereas sympathetic tone dominates during the day. This pattern suggests that parasympathetic dysfunction, rather than heightened sympathetic activity, underlies the autonomic impairment in patients with type 2 diabetes. The blunted nocturnal rise in HRV parameters and attenuated circadian variation across both time‐ and frequency‐domain measures, as confirmed by GAMM analysis, further support this interpretation. These findings align with prior reports, including Yu and Lee [20], which emphasises that parasympathetic dysfunction is an early and central feature of CAN in diabetes. Notably, cholinesterase inhibitor therapy, which enhances parasympathetic activity, has been associated with improved HRV by restoring autonomic balance and a reduced risk of cardiovascular events [21–23]. Given the parasympathetic impairment observed in stroke patients with type 2 diabetes, cholinesterase inhibitors may represent a promising therapeutic option in this high‐risk population.

In our study, although patients with type 2 diabetes exhibited relatively preserved circadian rhythms in both HR and HRV indices, the rhythmicity of HRV indices was notably attenuated, with reduced amplitude and less pronounced oscillations compared with HR. This differential pattern suggests that the regulatory mechanisms governing circadian HR and HRV are differentially vulnerable to diabetic complications. The preservation of circadian rhythmicity in HR and HRV in diabetes may indicate that the central circadian pacemaker in the suprachiasmatic nucleus remains functional, continuing to regulate HR and HRV through neurohumoral outputs and autonomic modulation [24]. Additionally, intrinsic cardiac mechanisms—such as the local molecular clock within cardiomyocytes—may contribute to the persistence of robust HR rhythmicity despite systemic metabolic disturbances [25]. Conversely, HRV is primarily influenced by the dynamic balance and reactivity of the autonomic nervous system, particularly parasympathetic (vagal) and sympathetic tone [26]. CAN, a common complication of diabetes, impairs both branches of the autonomic nervous system and growing evidence suggests that overall autonomic responsiveness is attenuated in diabetic individuals, even during circadian transitions [18]. This may account for the observed flattening of HRV rhythms. Taken together, these findings support the notion that diabetes differentially affects circadian cardiovascular regulation: while central circadian mechanisms governing HR and HRV remain relatively intact, the fine‐tuned autonomic control necessary for generating robust HRV oscillations is compromised, resulting in dampened but persistent circadian HRV patterns.

Although Perciaccante et al. [27] observed increased nighttime LF power in insulin‐resistant individuals with normoglycaemia, impaired fasting glucose or impaired glucose tolerance—suggesting early‐stage sympathetic overactivity—LF power was markedly reduced in patients with diabetes. Similarly, the present study demonstrated consistently lower LF and HF power in patients with type 2 diabetes across the 24‐h period, particularly characterised by blunted nocturnal variation (Figure 3). This reduction likely reflects diminished activity in both sympathetic and parasympathetic branches of the autonomic nervous system, consistent with more advanced stages of CAN, in which autonomic nerve damage impairs the normal modulation of HRV.

Elevated glycaemic levels have been linked to adverse effects on cardiac autonomic function in patients with diabetes. The most significant decline in HRV tends to occur in the early years following a diabetes diagnosis, particularly among those with poor glycaemic control [11]. In the present study, diabetic patients with suboptimal glycaemic control (HbA1c > 7% [53 mmol/mol]) exhibited higher HRs and lower HRV, especially during nighttime and early morning hours, compared to those with better control (HbA1c ≤ 7% [53 mmol/mol]). These time‐dependent differences mirrored those observed between diabetic and non‐diabetic individuals, albeit to a lesser extent, suggesting a dose–response relationship between glycaemic burden and autonomic dysfunction. These findings underscore the importance of avoiding sustained hyperglycaemia to preserve cardiac autonomic function.

Several limitations of this study should be acknowledged. First, it was conducted at a single centre with an ethnically homogeneous population, potentially limiting the generalizability of the findings to other ethnic groups or healthcare systems. In addition, because only patients with ischaemic stroke were included, we were unable to compare diabetic individuals with and without stroke; therefore, the stroke‐specific contribution to circadian autonomic alterations in diabetes could not be directly determined. Second, this was a secondary analysis of data originally collected for a different primary purpose, and the application of additional exclusion criteria may have introduced selection bias. Third, although the ECG patch provided 14 days of continuous monitoring, only the first 7 days were analysed to ensure data consistency, which may have led to underutilisation of the available data. Fourth, despite adjusting for a wide range of clinical and laboratory variables, residual confounding from unmeasured factors such as physical activity, sleep quality, psychological stress or medication adherence cannot be ruled out. Fifth, while HRV is a widely accepted surrogate for autonomic nervous system function, direct autonomic testing was not performed and could have provided more comprehensive physiological insight. Future prospective studies with multiethnic cohorts and direct autonomic testing are needed to confirm these findings and elucidate underlying mechanisms. Finally, pre‐stroke HR and HRV measurements were unavailable, as is common in acute stroke cohorts, and therefore causal attribution of stroke‐related or diabetes‐related changes in autonomic patterns cannot be definitively established. Future prospective, multiethnic studies incorporating direct autonomic testing are warranted.

## 5. Conclusions

This study underscores the substantial impact of type 2 diabetes and glycaemic control on circadian autonomic regulation in patients with ischaemic stroke. The observed alterations in HR and HRV reflect early autonomic impairment that may not be apparent through routine clinical evaluation. By leveraging continuous ECG monitoring, our findings provide novel insights into the time‐dependent nature of autonomic dysfunction associated with type 2 diabetes. Continuous autonomic monitoring may help identify nocturnal cardiovascular vulnerability in diabetic stroke patients. Interventions that enhance parasympathetic tone, including optimised glycaemic control and potentially cholinergic modulation, warrant further investigation as strategies to reduce post‐stroke cardiovascular risk. Together, these findings highlight the need for targeted approaches to monitor and manage autonomic function in stroke survivors.

## Author Contributions

Jiann‐Der Lee conceived and designed the study, curated the data, performed formal analysis and investigation, developed the methodology, supervised the project and draughted the original manuscript. Ya‐Wen Kuo contributed to study conceptualisation, formal analysis, investigation, funding acquisition, project administration and critical revision of the manuscript for important intellectual content. Yen‐Chu Huang and Meng Lee contributed to data curation, formal analysis, methodology development and investigation. Chuan‐Pin Lee contributed to data curation, formal analysis and investigation. Tsong‐Hai Lee contributed to methodology, formal analysis and investigation.

## Funding

This work was supported by grants from CHING PAO P.D. Charitable Foundation (Grant FCRPF6L0033).

## Disclosure

The authors remain fully responsible for the accuracy and integrity of the manuscript. All authors reviewed, edited and approved the final version of the manuscript for submission. Ya‐Wen Kuo is the guarantor of this work and, as such, had full access to all the data in the study and takes responsibility for the integrity of the data and the accuracy of the analysis. The sponsors played no role in the study design, data collection and analysis or decision to submit the article for publication.

## Ethics Statement

The study was approved by the Institutional Review Board of Chang Gung Memorial Hospital, Chiayi Branch, Taiwan (202101821B0C501). Patients provided written informed consent to participate in this study. All methods were carried out in accordance with relevant guidelines and regulations.

## Conflicts of Interest

The authors declare no conflicts of interest.

## Supporting Information

Additional supporting information can be found online in the Supporting Information section.

## Supporting information


**Supporting Information 1** Figure S1: Flow chart of patient selection.

**Supporting Information 2** Figure S2: Crude generalised additive mixed model analyses of circadian heart rate and heart rate variability patterns.

**Supporting Information 3** Figure S3: Stratified circadian patterns of heart rate and heart rate variability parameters according to stroke recovery stage.

**Supporting Information 4** Figure S4: Stratified circadian patterns of heart rate and heart rate variability parameters according to stroke severity category.

## Data Availability

The data that support the findings of this study are available upon request from the corresponding author. The data are not publicly available due to privacy or ethical restrictions.
