# Supplementary figures and images for: Type 2 Diabetes Intensifies Nocturnal and Early Morning Circadian Autonomic Dysregulation After Ischaemic Stroke
Source: J Diabetes Res. 2026 Apr 20;2026:4357436. doi: 10.1155/jdr/4357436 (PMC13095846; doi:10.1155/jdr/4357436)

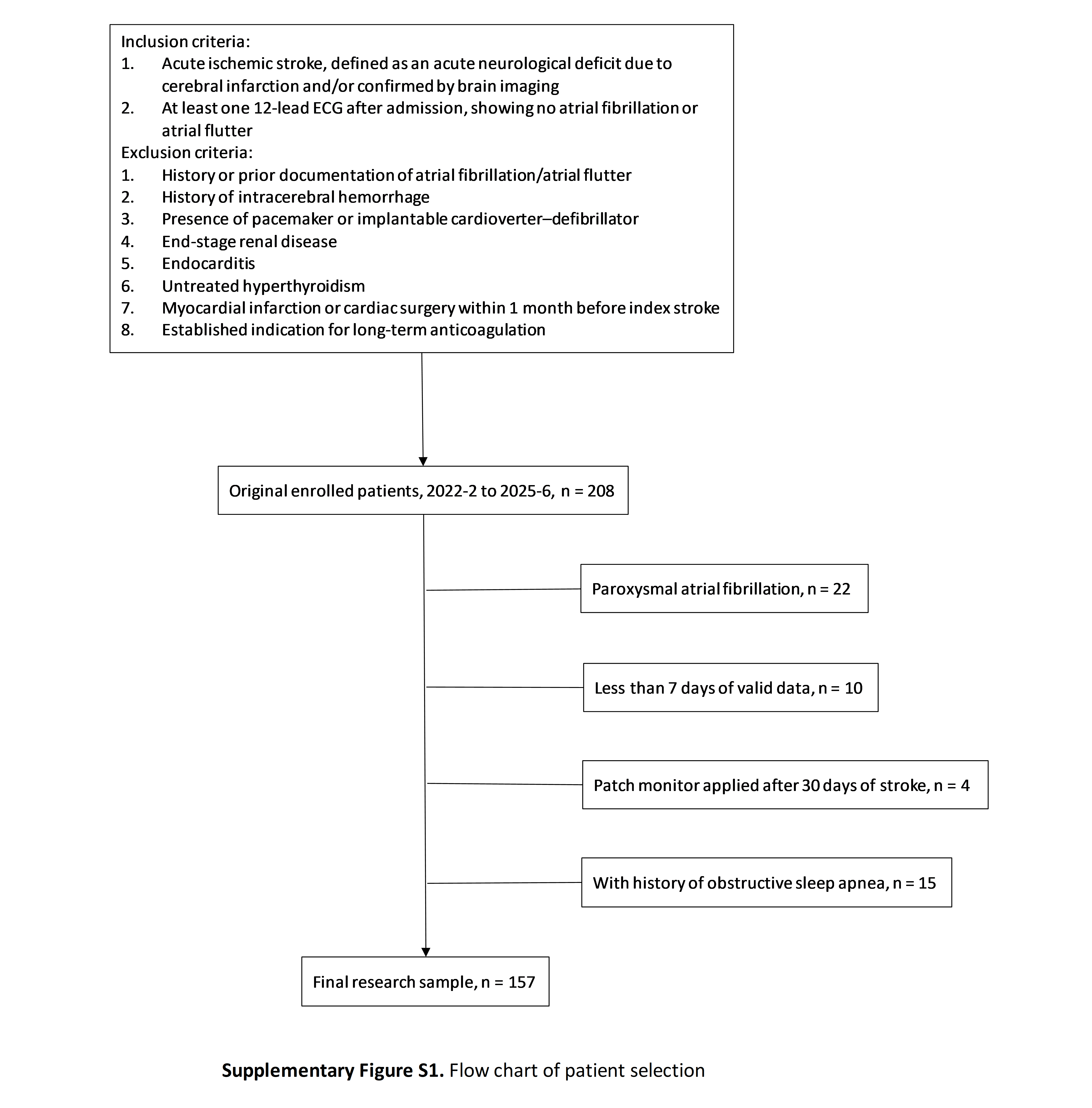

Supplement: Supplementary file 1 — Supporting Information 1 Figure S1: Flow chart of patient selection. [file JDR-2026-4357436-s001.tif]

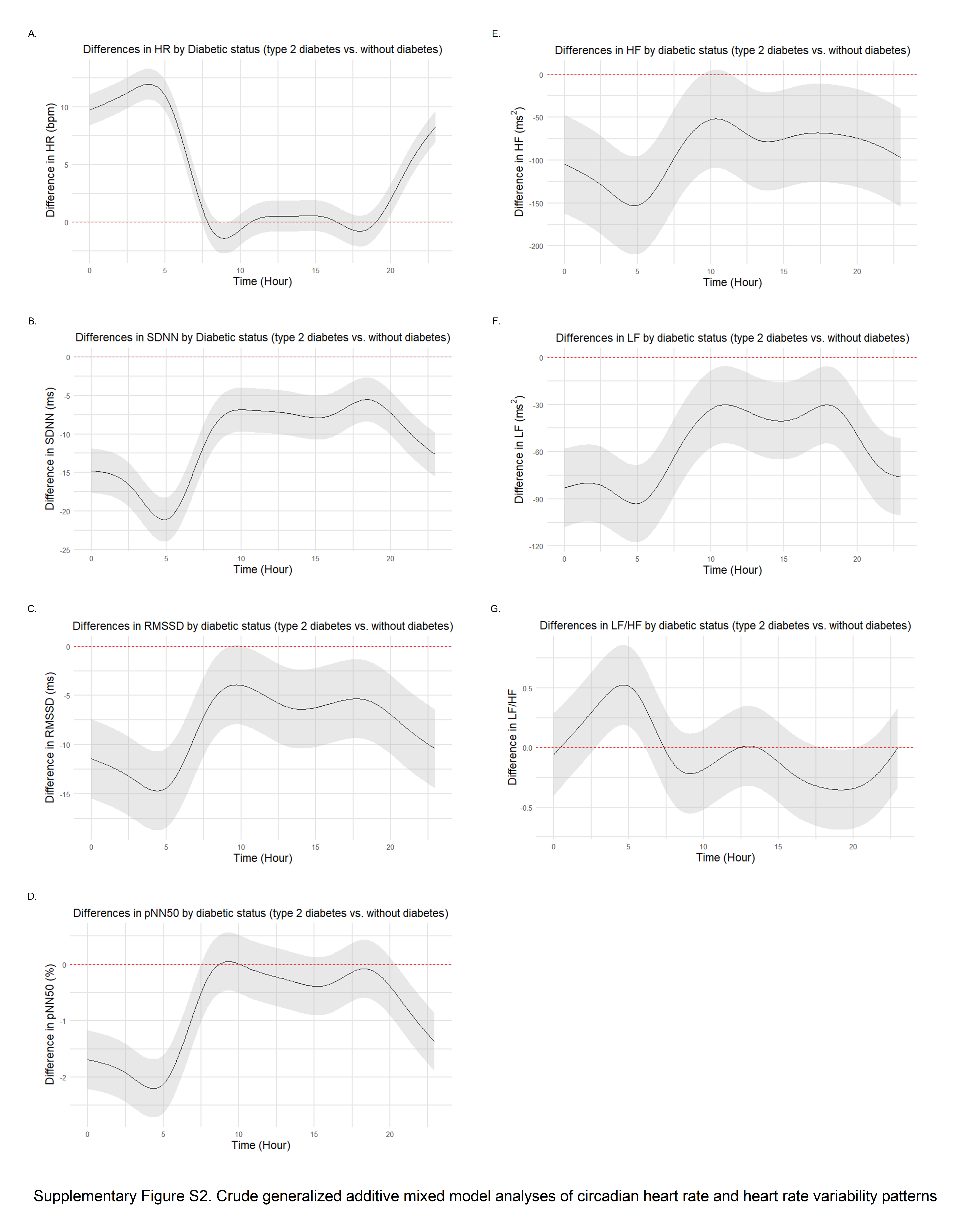

Supplement: Supplementary file 2 — Supporting Information 2 Figure S2: Crude generalised additive mixed model analyses of circadian heart rate and heart rate variability patterns. [file JDR-2026-4357436-s002.tif]

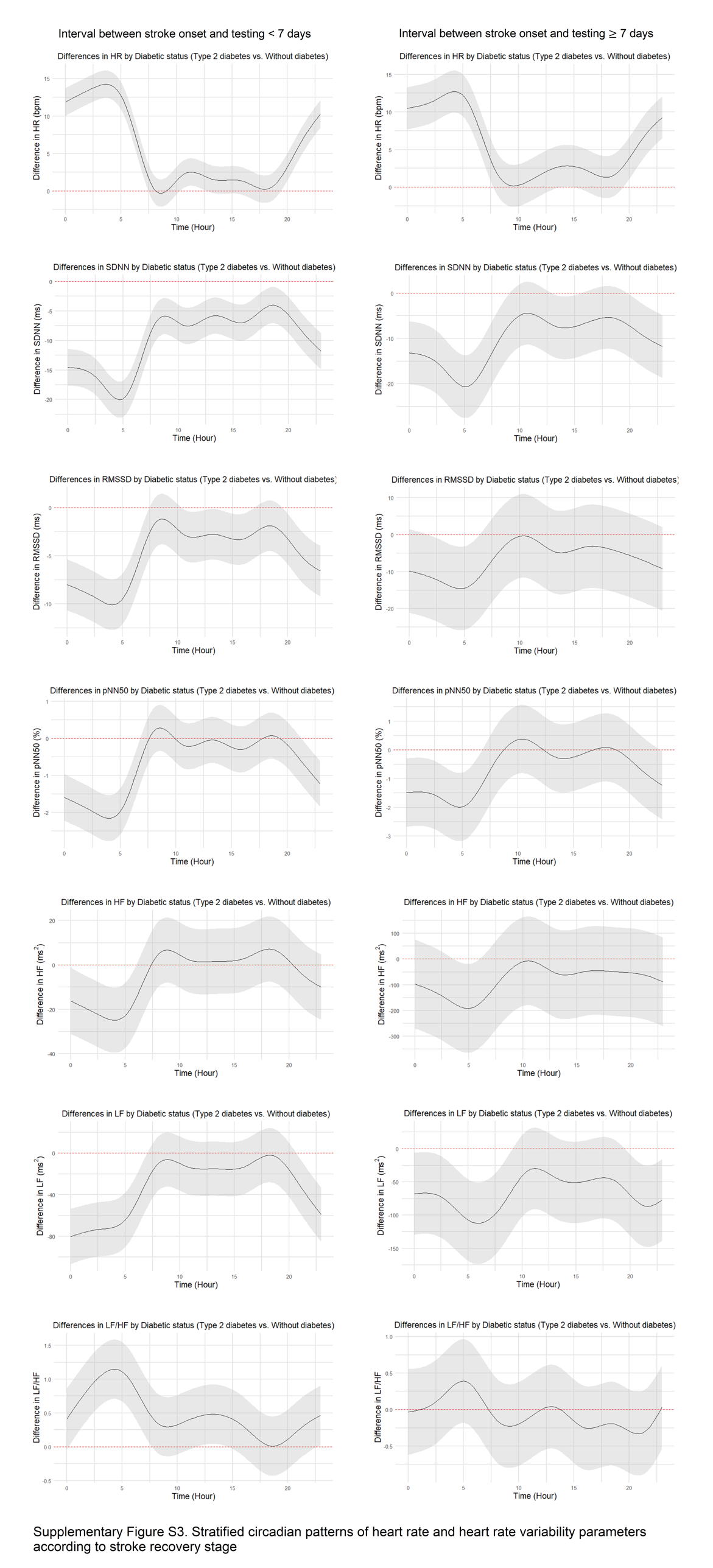

Supplement: Supplementary file 3 — Supporting Information 3 Figure S3: Stratified circadian patterns of heart rate and heart rate variability parameters according to stroke recovery stage. [file JDR-2026-4357436-s003.tif]

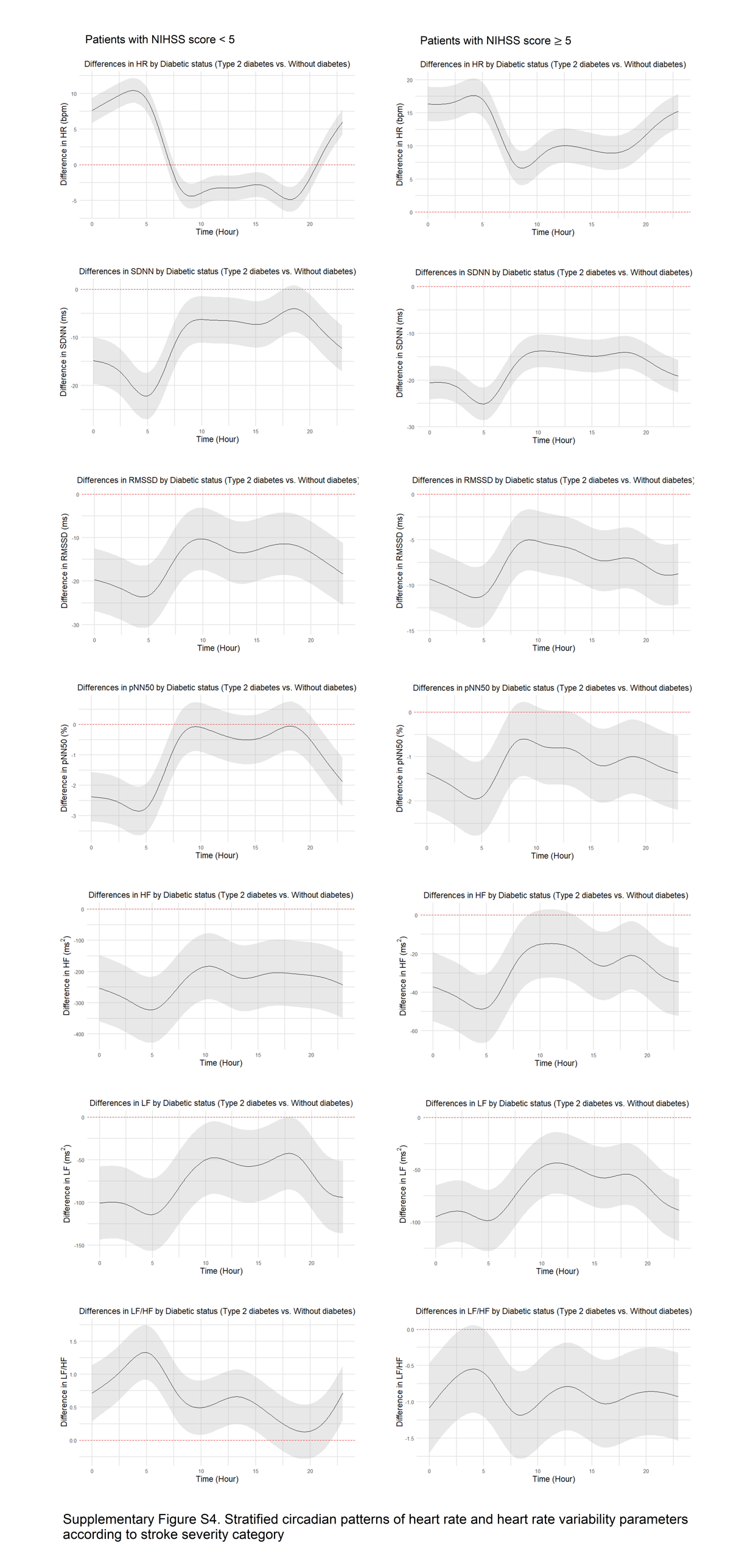

Supplement: Supplementary file 4 — Supporting Information 4 Figure S4: Stratified circadian patterns of heart rate and heart rate variability parameters according to stroke severity category. [file JDR-2026-4357436-s004.tif]
